# Supplementary material for: Acceptance and compliance with micronutrient powder (MNP) among children aged 6–23 months in northern Nigeria
Source: PLOS Glob Public Health. 2022 Oct 17;2(10):e0000961. doi: 10.1371/journal.pgph.0000961 (PMC10022258; doi:10.1371/journal.pgph.0000961)
Supplement: S1 Dataset — (ZIP) [file pgph.0000961.s008.zip › S8_Dataset/In-depth Interviews.zip!/In-depth Interviews/120715_A_GU_ID_2_CG.pdf]

In depth interview with caregiver in Kwadadai Community of Guyuk L.G.A, Adamawa State by Ishaku David Saraso. On Sunday 12 of July 2015.

Duration – 47m 38s

Introduction

I_ Describe for me the people I your immediate family living in your household.

R_ My name is Mai Anguwa Hussaini, I have two wife's which are Hadiza who is the first wife and sige is the second wife and my children's are as follows Hakuwnu, Ankiki, Godiya, Dauda, Pashent Salomi Musa, Nuhu, Solomon, Yuhanna, Mariya, Safiya. While Gambo, Goodluck and Barnabas are my **c**ousin but they are living in my house.

I Tell me about Children who age are between 6 – 23 months.

R_ We have Nuhu he is a male child and he is 7months old, children 6 – 23 months

CHILDREN 6 – 23 MONTHS

I_ We have heard of food challenges for young children in this setting related to the season.

R_ In the raining season foods like soya beans and bean prices use to rise because we don't crop or farm in our community most of time we buy them from the market so we don't feed the child much of beans and soya bean Pap and tea.

I_ You Mention that sometime the price of soya bean and beans rise which time

R_ During raining season the price use to rice

I_ Why?

R_ Because we have harvest the greens make used of some and sold some to the marketers. Most of the farmers use to buy it from the marketers to plant it in their farms and to cook.

I_What are the coping mechanism to these food challenges.

R_ I sell guinea corn that I harvested from my farm to buy soya beans and beans. I sell 10 majors of guinea corn to buy 2 majors of soya beans or beans. The challenge is what I support to eat with my family I have sold some to by that child food.

I_ How does this challenges affect infant and young child feeding.

R_ it does not affect infant and young child feeding alone but its also affect us as a family because it has reduce the food we support to eat with my family and to the infant and young child we reduce the food we use to feed him.

I_ One thing we heard in other interview that caregivers often give their children 6 – 23 month's tea. Could you define/explain tea?

R_ Since we are poor in this community we grand soya bean and bowled it and sieve the water and add sugar to it.

EXPERIENCE WITH MNP DURING TRIAL

I_ Now I want us to take about the MNP that you could you tell me about your experience with the produce.

R_ When MNP was given to my wife I was not at home when I came back she explain to me about the product and told me the name of the person Ali he is a community health worker. So when we gave the product to

the child after sometimes we notice changes in completion, changes in his health, he did not feel skin, not like before using MNP that he use to suffer diarrhea and fever he has also increase weight.

I_ You mention that it has changes his completion how?

R_ Changes in skin completion that I mention about is that when a child is not health you will see his skin looks scars but when we stat use MNP we notes some change in complex son of the child skin that shows that the child has improve in health.

I_ You also mentions that it has improved the child health how.

R_ Yes the child that is setting done in one place now that he is using MNP he is try to crow and whatever he want to do, he do with force that me that the produce is doing a very good job.

POSITIVE ASPECT OF USING MNP

I_ Tell me about any positive aspects of the experience with MNP.

R_To God be the glory because I am thinking of how to get the MNP agene to give my child agent when this one finish. I am happy because all my children that I have mention their names have not gotten that product which is given to my son Nuhu and is helping him so much that is why I am thinking of where to see you people to give us another MNP.

CHALLENGES/ BARRIERS USING MNP

I_ Tell me about any general challenges you had during the MNP trial.

R_ We have not seen any challenge before using the MNP and up to know there is no problem.

I_Is there any specific barriers that make it hard to use the MNP appropriately.

R_ There is no barriers in using MNP

I_ Some caregivers mentioned that is was hard to store the MNP without it being taken by others or eaten by rodents. Did you have any similar experience?

R_ No.

I_ Some mothers indicated that it might be hard to remember to use MNP did you have a similar experience and what did you do to remember?

R_ The only thing that made it hard to remember to use MNP is the time because you know that we are poor farmers most of the time our mind goes to the farm until we come back from the farm that we give the MNP to the child instead of given him in the morning we some time give him around 2pm but we don't forgot it for the holiday.

I_Tell me specific solutions that you suggests to address above mentioned challenge.

R_ The only solution I think is the way you come now to see us, there should be someone close to us that will advise us about the MNP and be checking whether that there are using it appropriately.

CHILD FEEDING BEHAVIOURS AND RESPONSE TO MNP

I_ let's talk about the child feeding and response to the MNP. How do you feed the MNP to the child usually?

R_ The way the mother usually feed the child (Nuhu) MNP, she usually prepare pap (kunu) and make it to be cool and fetch some like 3 to 5 spoons and mix with MNP and give to the Child.

I_ You mention that MNP was mixes with Kunu (pap) which type of kunu.

R_ We use to mix it with kunun dawa what we use to eat it is not all the time that we have soya to make kunu with or groundnut.

I_Now tell when the MNP was usually given to the Child.

R_In the morning around 6:00 am.

I_ Tell how extend does the child likes or dislikes the taste of the MNP.

R_ Yes the child likes the taste of the MNP.

I_Tell me stories/anecdotes which indicate that the child likes the Malp.

R_when the child (Nuhu) see his mother trying to mix the MNP with kunu he try to collect the cup from his mother.

I_Now tell me how MNP consumption impacted the child current diet.

R_The time that we state given him the MNP it has increased his appetizer and make him to eat food the more and all varieties of food.

SHARING MNP

I_ In other communities we noticed that sharing of MNP occurred. Could you talk about any sharing of the MNP when you used it?

R_I have notice that because when they brought MNP the say we should give it to Nuhu because they want to see how it will improve the child health we gave it to him along.

I_Tell me about sharing of MNP due to food shortage during lean season.

R_No we are not fasten because we are Christian.

I_ some caregiver are the MNP as a snack what will you say about that

R_ No

I_ Tell me whether 60 sachets will be enough for 60 day.

R_ Yes it will be enough for 60 day.

I_ Why

R_Because the MNP is one sachet per day that is why.

PERCEPTIONS OF THE MNP

I_ Now that you have used the MNP, could you talk about how you perceive it?

R_ The produce is very good for the children because it has help my child it improve his health and make him to be eaten food, increase in weight and change his completion.

I_ Can you tell me whether you see it more as food, a medicine or something else.

R_To me it is a food for children with medicine which improve my child health.

I_why do you sees it as food and medicine for children.

R_ Food aspect of it is that the way it is mix with kunu and feed the child and in the aspect of medicine when the act the mix kunu with MNP the way it is building the body of my child and also improve his health that is what makes me to see it as food and also medicine.

WILLINGNESS TO PAY FOR MNAP

I_ Now tells if MNP is cheaply on sale in this community will you be willing to buy.

R_ Yes

I_ Why?

R_ Because I have seen how it has help my child.

I_ Now that you know how it help your child, how much would you be willing to pay for each sachet.

R_You know in life no one don't like good health, so some of the medicine the hospital use to bring we know that same produce that is sole at N50 is more useful compare to the one sole at N20 but since I don't have enough money to buy that one that is sole at N50 I go for the ones sole at N20 but I know that the useful one is that one sole at N50 so that sachet I can buy at N50.

SOCIAL MARKETING THE MNP

I_You are given me great information now please tell me ways that you believe would be effective for promoting MNP to caregivers in the community so that they would comply with using it appropriately?

R_ My advise as Mai Anguwa is that when MNP will be share to community members they should follow the community heads to Mai Anguwa and since you people has gave MNP to 3 community members and have seen its benefits on the child let the child that has benefits from the MNP be shown to the women and the child has benefited be compare with the child that did not benefit let the caregivers see how it helps the child let them see their of improving his weight and also make him healthier. Tell them the time given up to now let them see the difference between his age mates to compare them when they see the difference the caregivers will ask for the MNP seen is believes.

I_ What way would be effective promote MNP for it appropriate use.

R_When sharing MNP the community health worker should call the man caregiver with the female caregiver and tell how to use the product and the use to the product to their child and the mail caregiver should be asked his wife whether she has given the MNP the child and if she say No the mail caregiver should feel her to give it to the child. This way will make both of them to be using the MNP appropriately.

I_ How are the people influence your opinions and knowledge about feeding practices?

R_ because it is community health work that brought the MNP, he knows what is good for our child, I know him and I know that it is his field of study that is what make me to use the MNP.

I_ Where would be good places to disseminate health information related to MNP.

R_ Let them come through the community heads and ward heads secondly let it be through religions leaders that will be the best way.

I_ who in your community do you trust the most for receiving messages about infant and young child feeding.

R_ it is the community health worker.

I_Tell me what of media do you prefer for receiving MNP messages.

R_ Prefer poster through the community heads and ward heads.

I_ What ways do you preferred of receiving education regarding IYCF and MNP.

I_ Tell me preferred ways of receiving education regarding IYCF and MNP

R_ They should come through village heads word head and community head and religions elders.

I_ Please tell me specific example of other successful health education campaigns in this community what made it successful?

R_ There are some immunization was done in the press that was not successful because refuse to collect it most of the Muslims they are thinking as if it will affect their productivity but when they come through the region leaders it will be successfully when imam health educated them it will be a success.

I_Tell me more about the one that was successful religions leaders were involves.

R_Their is one immunization of Ciwo Ina (Pollio) it was through the religions leader when the educate their members on the illness their effect and bout the immunization the community member agreed and receive it without any problem that is how it was successful.

I_ Please tells me best community channels to distribute the product.

R_ The produce should be through the community health workers so that they will explain to th4e district heads, village heads and ward heads the use of the produce.

I_ why do you prefer community health workers to distribute the products.

R_ The reason that I prefer community health work is because he knows about the produce, the dose and how supports to be given that product and all the community member know him and have trust in him. He is a staff with the government and the government use to send messages through him to the community members he also know how to share the MNP.

I_ Now I want to hear some specific messages you think would effectively promote the product.

R_ Let them look at the child has used MNP and the child that did not use MNP but there are of the same age and see the differences.

I_ Tell me effective messages to promote its use but limit the sharing of it.

R_ As a word leader, I can call out all the word members so that the community health worker will health educates them on the product. The health workers should also tell the caregiver that when the MNP is not use appropriately and limit the sharing.

FINAL THOUGHTS

I _ finally is there anything else that you can share about your experience with this product?

R_ I am happy because of the way it has help my son Nuhu.

I_ Could you tell me any recommendation to develop an effective program.

R_ I thank the government of Nigeria and also call on them to produce this produce very much so that it will be difficult for all parts of the country not only some parts to benefit from it.
